# Effects of high altitude on renal physiology and kidney diseases

**DOI:** 10.3389/fphys.2022.969456

**Published:** 2022-10-20

**Authors:** Si-Yang Wang, Jie Gao, Jing-Hong Zhao

**Affiliations:** ^1^ 953th Hospital, Shigatse Branch, Xinqiao Hospital, Army Medical University (Third Military Medical University), Chongqing, China; ^2^ Department of Nephrology, Shandong Provincial Hospital Affiliated to Shandong First Medical University, Jinan, Shandong, China; ^3^ Department of Nephrology, The Key Laboratory for the Prevention and Treatment of Chronic Kidney Disease of Chongqing, Chongqing Clinical Research Center of Kidney and Urology Diseases, Xinqiao Hospital, Army Medical University (Third Military Medical University), Chongqing, China

**Keywords:** high altitude, kidney, physiology, pathology, endocrine

## Abstract

The hypobaric and hypoxic conditions of high-altitude areas exert adverse effects on the respiratory, circulatory and nervous systems. The kidneys have an abundant blood supply (20%–25% of cardiac output) and high blood flow; thus, they are susceptible to the effects of hypoxia. However, the effects of acute and chronic exposure to high altitudes on renal physiology and pathology are not fully understood. Moreover, few studies have investigated the impact of high-altitude exposure on patients with chronic kidney disease or acute kidney injury. In this review, we summarized changes in renal physiology and renal pathology due to high-altitude exposure as well as the impact of high-altitude exposure on existing kidney diseases, with the aim of informing the prevention and treatment of kidney diseases at high altitudes.

## Introduction

High-altitude areas are common natural environments with extreme conditions, such as hypobaric, hypoxic and low-temperature environments. The atmosphere contains approximately 21% oxygen. However, high-altitude areas are characterized by atmospheric rarefaction; the atmospheric pressure and partial pressure of oxygen decrease with increased altitude. For example, at an altitude of 5,000 m, the partial pressure of oxygen is only half that at sea level. Therefore, even though the relative proportion of oxygen in the atmosphere remains constant, atmospheric rarefication can lead to hypoxia. Moreover, high-altitude exposure (i.e., hypobaric, hypoxic, and low-temperature conditions) can cause acute or chronic mountain sickness due to a lack of compensatory ability (Garrido et al., 2020). The series of alterations in metabolism and physiological functions due to high-altitude exposure impacts the health of people working and living at high altitudes. The effects of high altitude on the cardiovascular system, respiratory system and nervous system have been widely studied due to the prevalence of high-altitude heart disease (HAHD), high-altitude pulmonary edema (HAPE) and high-altitude cerebral edema (HACE). However, few clinical studies have focused on physiological and pathological changes in renal function at high altitudes. The influence of hypobaric and hypoxic conditions at high altitudes on renal function and structure as well as the progression of kidney diseases remains unclear.

Sodium transport is closely related to renal oxygen consumption (Swartz et al., 1977). Approximately 80% of total renal oxygen consumption is used to enable sodium reabsorption in the renal tubules, while the remaining 20% is associated with basic renal metabolism (Thurau, 1961). The complex vascular structure of the kidneys and the large oxygen demand imposed by solute reabsorption may make the kidneys more vulnerable to hypoxia (Fu et al., 2016). Blood flow in the renal medulla accounts for only 10% of the total renal blood flow, but approximately 30% of sodium chloride is reabsorbed in the medulla through an energy-dependent mechanism (Hansell et al., 2013). When the human body is exposed to high altitudes, changes in urine volume and blood pressure occur (Goldfarb-Rumyantzev and Alper, 2014). Different durations of high-altitude exposure exert different effects on renal function (Table 1). Short-term exposure produces acute responses of the acid-base balance as well as water and sodium excretion. However, long-term exposure gradually results in physiological adaptation, allowing renal function to normalize. The glomerular filtration rate and blood pressure also differ according to duration of high-altitude exposure. Additionally, the pathological characteristics of renal biopsy patients in plateau areas are different from those in plain areas (Zhou et al., 2014), indicating that the high-altitude environment may have an impact on renal function and structure. Hypoxia is an important risk factor for kidney diseases, and many kidney diseases occur upon exposure to varying degrees of hypoxia. For example, ischemia and hypoxia are important promoting factors in the occurrence and progression of chronic kidney disease. The impact of high-altitude exposure on kidney diseases such as high-altitude renal syndrome, acute kidney injury, chronic kidney disease and dialysis are discussed in this review, with the aim of informing the prevention and treatment of kidney diseases in high-altitude areas.

**TABLE 1:** Renal physiological response to acute and chronic high-altitude exposure.

	Response	Effects	
Acute exposure	Altered acid-base equilibrium	Increased pulmonary ventilation	Respiratory alkalosis
		Excretion of excess bicarbonate and retention of hydrogen ions	Compensation for alkalosis
	Circulatory changes	Increased excretion of sodium and bicarbonate	Increase in hematocrit
	Changes in the glomerular filtration rate (GFR)	Decrease in renal blood flow	Decreased GFR
	Endocrine changes	Increased levels of norepinephrine and adrenaline	Increased blood pressure
		Decreased RAAS activity	Decreased GFR
	Molecular changes	–	CD2AP, nephrin, EPAS1, EGLN1, PPARα and PHD2
Chronic exposure	Circulatory changes	–	Increased hemoglobin levels and blood viscosity
	Changes in the glomerular filtration rate (GFR)	Increased renal hyperfiltration and filtration fraction	Increased GFR
	Endocrine changes	Increase in adrenaline levels	Increased blood pressure
		Increased RAAS activity	Increased renal hyperfiltration
	Molecular changes	–	HIF-1α, HIF-2α, VEGF and VEGFR-2

CD2AP, CD2-associated protein; PHD2, prolyl hydroxylase 2; RAAS, renin–angiotensin–aldosterone system.

High-altitude exposure also affects endocrine function, including impacts on the sympathetic nervous system (which produces adrenaline) and the renin–angiotensin–aldosterone system (RAAS). During adaptation to high altitudes, endocrine changes mainly enable more effective use of oxygen by tissues and organs and reduce the adverse effects of hypoxia. Erythropoietin (EPO) is an important regulator of renal hypoxia. In the early stages of adaptation, the kidneys increase excretion of salt and water, resulting in a higher concentration of red blood cells and increasing the hematocrit level. This alteration indirectly increases the oxygen-carrying capacity of blood. The continuous decline in oxygenation of the renal cortex and medulla drives a chronic adaptive response and stimulates the expression and synthesis of EPO (Donnelly, 2001).

The purpose of this study was to summarize the physiological changes in the kidneys after the acute and chronic high-altitude exposure as well as the effect of high-altitude exposure on the kidneys under pathological conditions. We also clarify the roles of kidney-related hormones under altitude-induced hypoxia. We hope this review provides new insights and suggestions for protecting kidney health in people at high altitudes.

## Physiological changes

### Acute exposure

In response to acute high-altitude exposure, the body increases tissue oxygenation by increasing pulmonary ventilation, contributing to respiratory alkalosis (Goldfarb-Rumyantzev and Alper, 2014). The kidneys compensate for this alkalosis by excreting excess bicarbonate and retaining hydrogen ions to reduce respiratory alkalosis while maintaining the increased oxygenation (Luks et al., 2008). Exposure to hypoxic conditions also increases renal excretion of sodium and water, resulting in a reduction in circulating levels of sodium and water and a corresponding increase in hematocrit levels, which can offset the reduction in oxygen supply. At higher altitudes (1,700–2,800 m), urine pH levels can also rise (Ge et al., 2006). Therefore, higher altitudes have a diuretic effect. This natriuretic effect, in addition to the diuretic effect of bicarbonate, may be one of the reasons for the increase in urine output. A decrease in antidiuretic hormone levels has also been observed (Bestle et al., 2002; Haditsch et al., 2015). However, the effect of high-altitude exposure duration on diuresis and the underlying mechanism remain unclear. The diuretic effects can increase the concentration of red blood cells, which may be the basis for the initial increase in hemoglobin and hematocrit concentrations (Bestle et al., 2002; Haditsch et al., 2015).

Changes in renal metabolism can also cause changes in the glomerular filtration rate (GFR), which is used to measure renal function. However, studies on the GFR after short-term exposure to high altitudes have yielded inconsistent findings. Two studies found no significant change in the GFR after 8–12 h or 48 h of exposure to an altitude of 4,000–5,000 m (Jefferson et al., 2002; Loeppky et al., 2005). However, another study reported that the GFR decreased as altitude increased (at 4,500 m) (Pichler et al., 2008). Similarly, the GFR was found to decrease after high-altitude exposure, showing a significant decrease on day three (at 4,500 m) (Bestle et al., 2002). This discrepancy may be related to the initial red blood cell concentrations and the decrease in renal blood flow. Controversial results were also obtained when inulin clearance, creatinine clearance or the serum creatinine level were used to estimate the GFR under conditions varying in hypoxia. Because exogenous markers, such as inulin or isotopes, cannot be evaluated at high altitudes, the ability to assess renal function may be limited (Haditsch et al., 2015).

**FIGURE 1:**
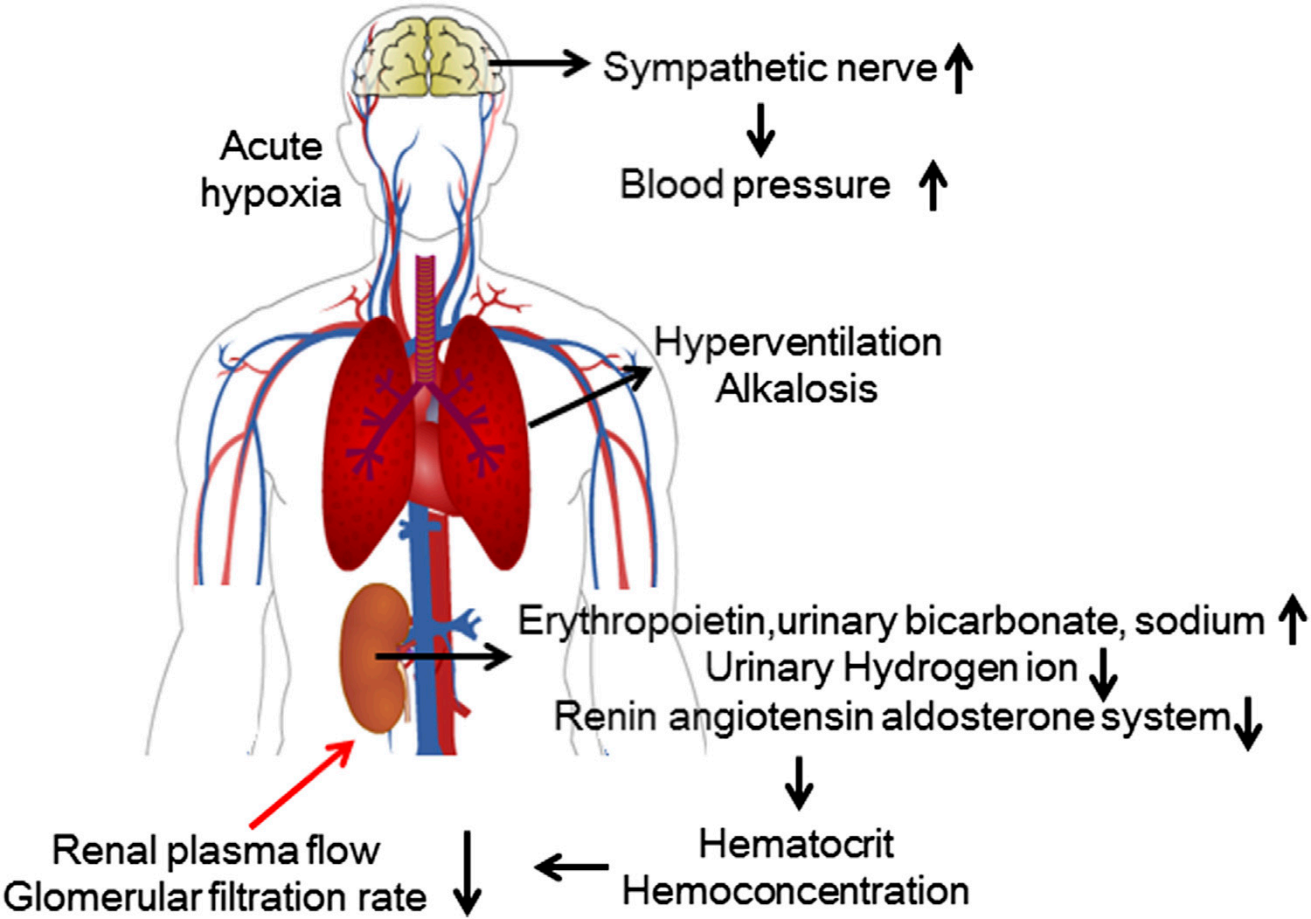
Renal physiological response to acute high-altitude exposure. During acute exposure to hypoxic conditions, activation of the sympathetic nervous system causes elevated blood pressure. The kidneys compensate for hyperventilation-induced alkalosis by regulating electrolyte metabolism. The compensatory increase in red blood cells leads to a relative decrease in renal blood flow, and the suppression of RAAS activity leads to a decrease in the GFR.

High-altitude hypertension is a common symptom of mountain sickness. During acute and chronic exposure to high altitudes, blood pressure (BP) is elevated; however, the mechanism underlying this elevation may differ. During acute high-altitude exposure, the increased BP may be partly mediated by activity of the sympathetic nervous system. In one study of acute exposure to high altitude (at 4,500 m), BP began to rise on days 1–3, but norepinephrine levels in the blood did not increase significantly until day 6 (Bestle et al., 2002). In another study, systolic BP and epinephrine levels increased on the first day of exposure to high altitude (at 4,500 m); subsequently, diastolic BP and mean BP increased with increases in the norepinephrine concentration (Kanstrup et al., 1999). The rapid and powerful diuretic effect induced by the hypobaric and hypoxic conditions of high-altitude exposure (at 1,500–5,500 m) is accompanied by a decrease in circulating levels of antidiuretic hormone, renin and aldosterone as well as an increase in natriuretic hormone levels and plasma and urinary adrenaline levels (Goldfarb-Rumyantzev and Alper, 2014) (Figure 1).

After short-term exposure to high altitude, the moleculers and protein expression in the kidney undergo adaptive changes. After rats spent 14 days in a simulated high-altitude environment (5,000 m), electron microscopy showed podocyte injury in their kidneys. The protein levels of CD2-associated proteins and nephrin in the glomeruli were lower than those in the control group, possibly related to proteinuria after short-term high-altitude exposure (Zeng et al., 2022). NO is also a component of the hypoxia response. During short-term, intermittent, hypobaric and hypoxic conditions, enzymes related to the urea cycle in the liver, brain and kidney maintain NO homeostasis (Javrushyan et al., 2021). After rats underwent 5 days of exposure to altitudes of 3,400 m or 4,300 m, the expression of high-altitude adaptability genes, such as EPAS1, EGLN1, PPARα and oxygen sensing protein prolyl hydroxylase 2, in their kidneys was significantly higher than that in those kept at sea level (Xie et al., 2015).

### Chronic exposure

Several studies have shown that long-term exposure to high altitudes (ranging from 2,800 to 5,800 m) increases hematocrit levels and that this increase is accompanied by increases in hemoglobin levels and blood viscosity (Thron et al., 1998; Singh et al., 2003; Al-Hashem et al., 2012; Zouboules et al., 2018). The GFR undergoes similar changes. After chronic high-altitude exposure, the GFR will eventually stabilize. For example, the GFR was similar to baseline on day 7 at 4,500 m (Bestle et al., 2002). Similarly, the GFR decreased on day 3 at 3,440 m but remained stable by day 14 at 5,050 m (Haditsch et al., 2015). This pattern may be related to increased renal blood flow. Because the increase in hemoglobin levels increases blood viscosity and renal blood flow, this may be a response to the increase in renal vascular resistance, as found at 5,000–6,000 m (Thron et al., 1998). Renal plasma flow (RPF) and the glomerular filtration percentage of RPF (filtration fraction, FF) are the main factors that determine the GFR. A typical FF value is approximately 20%. In patients with high-altitude polycythemia (HAPC), RPF decreased significantly due to the increase in hematocrit. However, the GFR remained relatively unchanged due to the increase in the FF(Hurtado et al., 2012). For example, a study showed that the FF of men living at sea level was approximately 18%, while that of men with moderate polycythemia living at high altitudes was 25%. The FF of men with severe polycythemia and chronic mountain sickness living at high altitude (at 5,000–6,000 m) was 28% (Lozano and Monge, 1965).

**FIGURE 2:**
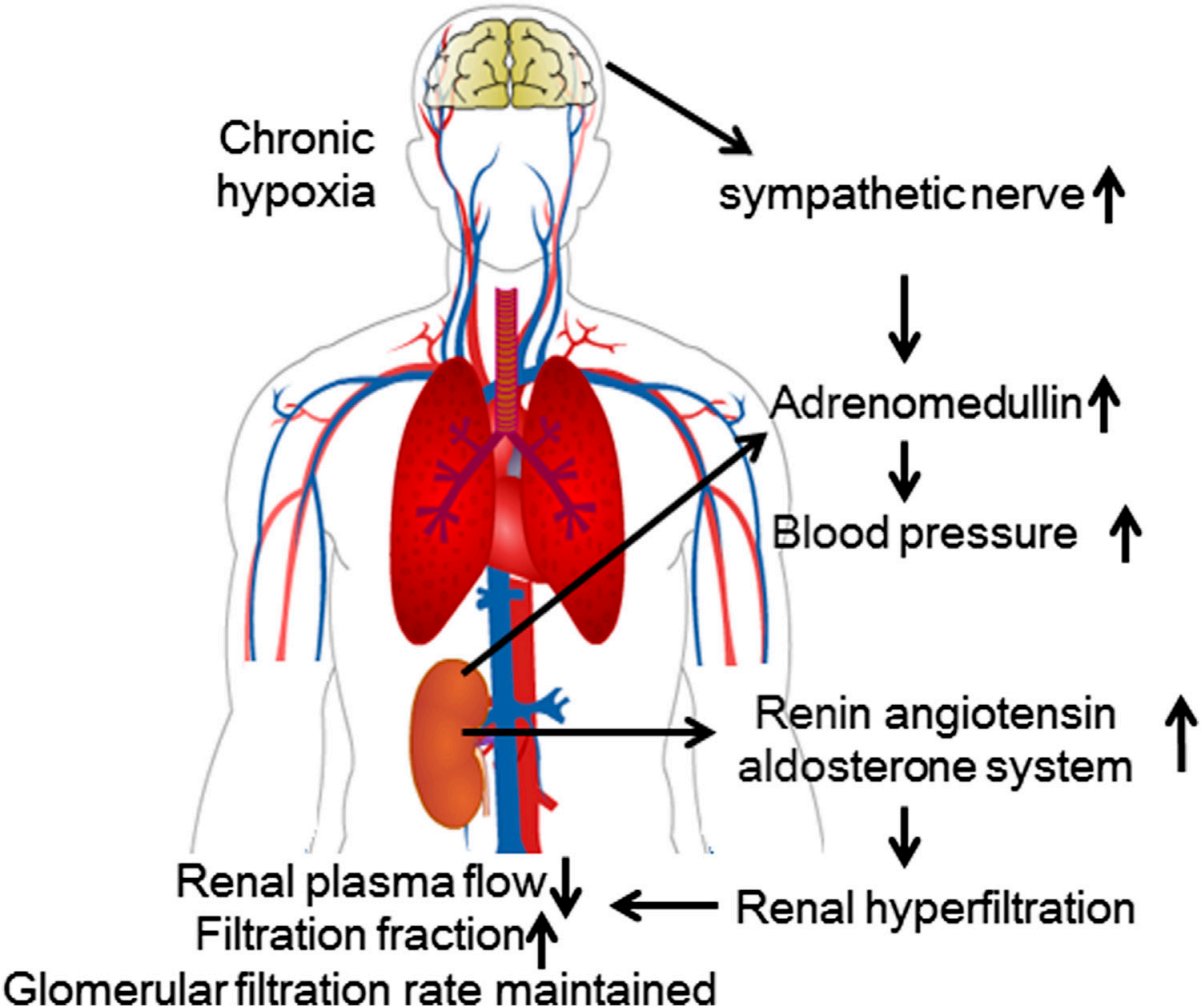
Renal physiological response to chronic high-altitude exposure. During chronic exposure to hypoxic conditions, the increased activity of the RAAS causes glomerular hyperfiltration. Although renal blood flow decreases, the GFR remains relatively unchanged.

BP increases when individuals undergo chronic high-altitude exposure. This increase may depend on erythrocytosis. However, the relationship between levels of regulatory hormones and BP is not clear. RAAS activity and levels of norepinephrine and vasopressin may be important for the increase in mean arterial pressure during long-term exposure to high altitudes (Palubiski et al., 2020) (Figure 2).

For long-term high-altitude residents, higher altitudes were associated with worse kidney function. Altitude was negatively correlated with the GFR when only urban areas were analyzed. At high altitudes (2,300–3,800 m), there was a trend toward a negative association between hemoglobin levels and the GFR (Carrillo-Larco et al., 2019). A lower GFR may facilitate reductions in reabsorption and oxygen consumption. Furthermore, people who live at high altitudes (at 3,600 m) have a higher prevalence of proteinuria and a lower prevalence of metabolic syndrome (Hurtado-Arestegui et al., 2018).

Regarding changes in gene expression after chronic high-altitude exposure, research has mostly been conducted with plateau wildlife. A transcriptome study of multiple organs in yaks living at 3,400 m, 4,200 m, and 5,000 m, revealed that the number of genes related to hypoxia in the differentially expressed genes of kidney accounted for 6.25%, second only to those in the heart (Qi et al., 2019). A comparison of the kidneys of Tibetan sheep, plains sheep, and goats revealed that the expression of HIF-1a, HIF-2a, VEGF and VEGFR-2 in the renal tubules of Tibetan sheep was significantly higher than that of ordinary sheep and goats (Yang et al., 2020). The above proteins are important regulatory proteins for adaptation to high-altitude hypoxic environments.

## Pathological changes

### High-altitude renal syndrome

In 2011, Hurtado et al. proposed the concept of high-altitude renal syndrome (HARS), which consists of high-altitude polycythemia, hyperuricemia, systemic hypertension and microalbuminuria. Pulmonary hypertension is considered to be another feature of HARS (Arestegui et al., 2011).

The prevalence of proteinuria is higher among populations living at high altitudes. As early as 1987, an observation of 14 healthy people showed that proteinuria increased 4–6 days after exposure to an altitude of 4,846 m (Winterborn et al., 1987). A study reported that 15% of Tibetans had microalbuminuria (Chen et al., 2011). The pathogenesis of proteinuria may be related to many factors, including the effect of hypoxia in renal parenchyma, glomerular hypertension, high blood viscosity and increased right heart pressure.

Proteinuria is also associated with hyperuricemia, which is common at high altitudes. Hyperuricemia in populations living at high altitude was first reported in 1968 (Sobrevilla and Salazar, 1968). Recently, a study found that the prevalence of hyperuricemia among 4,198 employees who lived in high-altitude areas was 28.1% (Shen et al., 2019). In contrast, the prevalence of hyperuricemia reported in the general Chinese population is 8.4–13.3% (Liu et al., 2015). This discrepancy may be related to the decrease in ATP levels with the increase in adenine nucleotide turnover and the activation of xanthine oxidase. In addition, lactate produced under hypoxic conditions competes with urate excretion in proximal tubules, resulting in decreased urate clearance. Polycythemia also leads to elevated blood uric acid levels due by cell renewal (Arestegui et al., 2011). Multivariate analysis showed that hyperuricemia, polycythemia and hypertension were independent predictors of albuminuria (Chen et al., 2011). Although HARS was proposed 10 years ago, few studies have focused on the specific mechanism underlying it. Recently, Zhao et al. published a study on the pathology of HAPC complicated with proteinuria (Wang et al., 2022). They defined the combination of HAPC and symptoms of kidney damage as HAPC-related nephropathy. Symptoms of renal injury may be abnormal values on routine urine tests and/or decreases in the estimated GFR. Through analysis of the clinical indicators and pathology of HAPC in patients who underwent renal biopsy, Zhao found that patients with HAPC-related kidney disease exhibited several main histopathological features: glomerular hypertrophy, basement membrane thickening, effacement of podocyte foot processes and segmental or global glomerulosclerosis. However, not all of these patients met the criteria for HARS.

### Chronic kidney disease

A 2011 epidemiological study of CKD in a Tibetan population found that the prevalence of CKD and the related proteinuria, hypertension and hyperuricemia incidence rates among high-altitude residents were much higher than those in individuals living in low-altitude areas such as Beijing and Guangzhou (Chen et al., 2011). Elevated blood pressure caused by chronic hypoxia, increased cell proliferation, increased collagen synthesis, endothelial cell dysfunction (Sánchezlozada et al., 2008) and increased uric acid production during hypoxia (Kang et al., 2002) as well as genetic factors and dietary structure may all be involved in the occurrence of CKD at high altitudes (Arestegui et al., 2011). Multivariate logistic regression showed that age, female sex, systolic blood pressure, fasting blood glucose, and primary school or lower education were associated with a higher risk of CKD (Zhang et al., 2018). Therefore, CKD patients with long-term high-altitude exposure may exhibit faster progression to ESRD than those who live at sea level. In a comparison of 369 patients with biopsy-confirmed diabetic nephropathy (DN), patients living ≥2,000 m above sea level had higher mean body mass, hemoglobin concentrations, and baseline GFR than those living at lower altitudes. During the 20-month follow-up, 38% of the patients progressed to ESRD. In a multivariate Cox analysis, living at high altitudes was independently associated with progression to ESRD in Chinese DN patients. (Zhao et al., 2020).

The increased risk of volume overload in hemodialysis patients may lead to pulmonary edema and arterial hypoxemia. Dialysis-dependent patients who stayed at a median altitude of 2,000 m for 2 weeks gained more weight than those who stayed at an altitude of 576 m during dialysis (Mairbäurl et al., 1989). A 20-year-old man with CKD undergoing peritoneal dialysis (PD) developed dyspnea and pulmonary congestion after visiting a high-altitude city (3,827 m above sea level). CKD and PD may be risk factors for the development of high-altitude pulmonary edema due to pulmonary hypertension and fluid overload (Vizcarra-Vizcarra and Alcos-Mamani, 2022). Observation of the mineral and bone disorder (MBD) status of hemodialysis patients in multiple centers in Tibet indicated that their MBD status was far from ideal. High altitude maybe a possible explanation of this result (Dang et al., 2019). In a study in Peru, dialysis at high altitude did not increase patient mortality, but patients with DN who received dialysis had significantly higher mortality (Bravo-Jaimes et al., 2021). Due to impaired erythropoietin production and shortened erythrocyte survival time in patients with CKD, there is no expected erythropoietin response to high altitude. There was little change in HCT, reticulocyte or erythropoietin production in people who stayed at high altitudes (between 2,000 and 4,600 m) for more than 2 weeks. Because the poor hypoxic ventilation response in high-altitude areas may make individuals prone to acute mountain sickness (AMS), mild metabolic acidosis may have a protective effect. However, some CKD-induced changes may increase the risk of altitude sickness. Anemia reduces oxygen delivery and predisposes people to AMS. In addition, metabolic acidosis can enhance pulmonary vasoconstriction (Luks et al., 2008). The specific altitude, oxygen concentration and exposure duration may all affect the results of studies. Another study showed that CKD increases the risk of thrombosis in travelers at high altitudes. Low-molecular-weight heparin is useful only for travelers at high risk of venous thromboembolism. Time differences and circadian rhythm disorders can increase cardiovascular disease events in CKD patients (Furuto et al., 2020). Patients with CKD who remain at high altitudes are at increased risk of CKD progression, altitude sickness, and pulmonary edema progression (Goldfarb-Rumyantzev and Alper, 2014).

### Acute kidney injury

Few epidemiological studies have investigated AKI in populations at high altitudes. At present, the impact of the high-altitude environment on AKI is not clear. Zhu et al. reported a case of AKI. A healthy young man developed AKI after sudden exposure to high altitudes (up to 5,200 m). On the fourth day at this altitude, he began to vomit and was flown back to sea level the next day. His blood urea nitrogen level was 8.8 mmol/L, and his serum creatinine (Cr) level was 319 μmol/L. The patient had already entered the diuretic phase and was in a stable condition. He was administered only supportive care, including alprostadil and reduced glutathione. His serum creatinine level decreased to 97 μmol/L after 35 days. Although the specific causes of AKI were not identified, the authors suggested that acute systemic hypoxia and long-term renal hypoperfusion may have been the causes of renal injury (Yijiang et al., 2013). In another study, high altitude was suggested to have an adverse influence on hypertensive disorders of pregnancy-related AKI, with earlier termination of pregnancy and a higher likelihood of stillbirth/neonatal death (Li et al., 2021).

**FIGURE 3:**
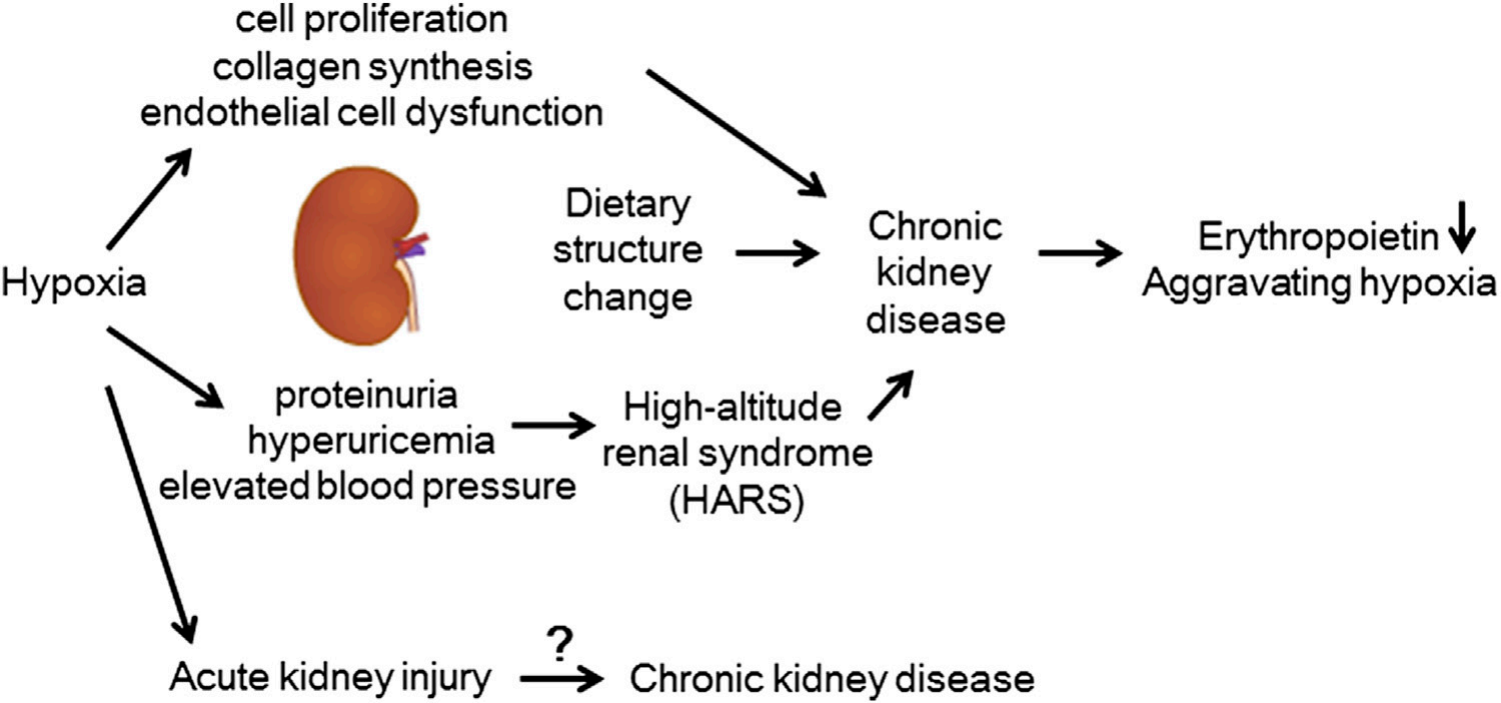
Kidney disease at high altitudes. Elevated blood pressure caused by chronic hypoxia, increased cell proliferation, increased collagen synthesis, endothelial cell dysfunction, increased uric acid production, and dietary structure may all contribute to the occurrence of CKD at high altitudes.

The traditional view is that treatment can restore the renal function of AKI patients to the level before condition onset, but recent large-scale clinical studies have shown that the occurrence of pathological repair, such as chronic fibrosis, at follow-up in AKI patients greatly increases the potential risk of CKD occurrence and progression and may even cause them to directly progress to ESRD and require dialysis or transplantation. In recent years, an increasing number of studies have shown that AKI and CKD are two closely related clinical syndromes (Chawla et al., 2014). Therefore, effectively delaying the progression of AKI to CKD is an important method to reduce the incidence rate of CKD. Renal interstitial fibrosis is a major marker of CKD and is also considered a predictor of disease progression (Webster et al., 2017). At present, it remains unclear whether a high-altitude hypobaric, hypoxic environment can aggravate the occurrence and development of renal fibrosis in the course of AKI and accelerate the chronic progression to CKD as well as whether reducing the high-altitude exposure duration of AKI patients. Although high-altitude exposure increases the incidence rate of CKD, the mechanism has not been fully clarified, and the effect of high-altitude exposure on renal function and structure in the pathological state is not clear. Therefore, it is very important to clarify the impact of high-altitude exposure on the progression of AKI to CKD to improve clinical diagnosis and treatment (Figure 3).

## Endocrine changes

### Adrenomedullin

At high altitude, an increase in sympathetic activity is manifested by an increase in the urinary norepinephrine concentration. The sustained increase in sympathetic activity may explain the downregulation of adrenergic receptors after prolonged exposure to high altitudes (Ponchia et al., 1994). Hypoxia directly stimulates adrenaline release from the adrenal medulla, resulting in an increase in the circulating concentration. The degree of this release depends on the degree and severity of hypoxia, and the decrease in arterial oxygen content is the main stimulus. Urinary adrenaline excretion increases significantly after acute high-altitude exposure (Mazzeo et al., 1994; Mazzeo et al., 1998) and then returns to the concentrations at sea level after the first week, with adaptation (Mazzeo and Reeves, 2003). The result is increased heart rate, stroke volume, tissue vasodilation, and bronchiectasis, all of which increase oxygen delivery to tissues.

### The renin–angiotensin–aldosterone system

The impact of high altitude on the RAAS has been studied for more than 50 years (Ayres et al., 1961). However, the changes in the RAAS caused by short-term exposure to high altitude are still controversial (Keynes et al., 1982). It is widely accepted that renin activity and aldosterone levels are decreased after a high-altitude exposure of several days to 1 month (Palubiski et al., 2020). Regarding the decrease in aldosterone levels, some studies suggest that it may not be closely related to angiotensin-converting enzyme (ACE) levels because ACE levels do not change significantly after short-term high-altitude exposure (Cooke et al., 2018). The increase in aldosterone levels may be attributed to the elevated levels of adrenomedullin observed at high altitude (Taylor and Samson, 2002) or the downregulation of adrenal angiotensin II receptors (Chassagne et al., 2000). The effect of high-altitude hypoxia on plasma levels of RAAS-related hormones may depend on the duration of exposure to high altitude, the elevation, and physical activity. Acute exposure to hypoxic conditions can cause a decrease in ACE concentration, which may protect against an increase in plasma aldosterone and angiotensin II levels. This decrease could lead to severe vasoconstriction and sodium retention and may be one of the reasons for the increased excretion of water and sodium, which may be beneficial for reducing the occurrence of edema. No clinical study has focused on the effect of long-term high-altitude exposure on the RAAS. In a basic study, the levels of RAAS-related hormones in rats increased after 90 days of high-altitude exposure. Activation of the RAAS promotes glomerular hyperfiltration through efferent arteriolar vasoconstriction (Helal et al., 2012).

### Erythropoietin

The production of EPO can be used to increase the mass of red blood cells as well as the hemoglobin concentration to improve the oxygen-carrying capacity of blood (Faura et al., 1969). EPO levels can increase with increasing altitude. At altitudes above 2,000 m, EPO production increases, and it remains high after 24 h. EPO peaks at 24–48 h of high-altitude exposure and decreases to baseline within a few weeks with the increase in hematocrit concentration and feedback inhibition (Lehtiniemi et al., 2005). However, in low-altitude areas, the change in EPO is transient and will not last beyond 24 h of exposure.

Individuals with ESRD living at high altitudes may better respond to endogenous and exogenous EPO. EPO responsiveness is negatively correlated with mortality risk. Hypoxia is also considered to affect EPO responsiveness. A retrospective cohort study of patients with ESRD found that people living at high altitudes (>2,000 m) needed a smaller dose of EPO to maintain a higher hematocrit level. The resistance to EPO decreased with increasing altitude (Brookhart et al., 2008). Similar results were found by comparing MHD patients in Tibet (3,650 m above sea level) and Beijing (43.5 m above sea level). Hypoxia at high altitude improved EPO responsiveness in MHD patients. A comparison of the weekly weight-adjusted EPO dose divided by the hemoglobin concentration revealed that the erythropoietin resistance index of the high-altitude group was lower than that of the low-altitude group (Wang et al., 2021).

### Remedial measures and management

At present, effective treatments and measures of the effects of high altitude on renal physiology and pathology are lacking. The most effective measure to eliminate these effects is to reduce the altitude of the patient. When it is impossible to reduce the altitude, continuous oxygen inhalation to achieve oxygen saturation that is basically consistent with that of the plain may be important to alleviate the adverse effects of hypoxia on the kidney. Hyperbaric oxygen is a treatment for AMS, but there is no research on whether hyperbaric oxygen can alleviate the effect of chronic high-altitude exposure on the kidney. Acetazolamide may alleviate pathological changes in the kidney, especially proteinuria, by increasing arterial oxygen levels within a short time. For example, recent research has shown that acetazolamide can treat focal segmental glomerulosclerosis secondary to high-altitude polycythemia (Vizcarra-Vizcarra et al., 2022). Moreover, the use of angiotensin-converting enzyme inhibitors or angiotensin receptor blockers (ACEIs/ARBs) has been recognized as an effective measure to reduce microalbuminuria and reverse ventricular remodeling. Therefore, treatment with ACEIs/ARBs may be beneficial for reducing the pathological changes in the heart and kidney at high altitudes. The Lancet has reported a prospective study on ACEIs in the treatment of HAPC. ACEIs can significantly reduce the levels of proteinuria and hemoglobin in high-altitude residents with HAPC and 24-h urinary protein excretion greater than 150 mg (Plata et al., 2002). Adjustment of medication after entering high altitude is not recommended for most patients with CKD or DN, but they should closely monitor blood pressure, blood glucose, urine volume and other indicators. In addition, the use of NSAIDs should be avoided as much as possible. (Luks et al., 2008).

## Conclusion

The effect of high-altitude exposure on the kidneys is obvious. Acute and chronic exposure to high altitudes has different effects on renal physiology. Among the pathological changes, polycythemia, hyperuricemia, systemic hypertension, and microalbuminuria are strongly correlated with high-altitude exposure. High-altitude exposure can promote the progression of CKD to ESRD as well as additional complications, and people with CKD have an increased risk of AMS at high altitudes. While research on the changes in AKI at high altitudes is lacking, a high-altitude hypoxic environment may be an important factor in the prognosis of AKI and the progression of AKI to CKD. Reducing the altitude and oxygen inhalation may be the best measures to alleviate the effect of high-altitude exposure on the kidney. The use of ACEIs/ARBs is still necessary and effective for the treatment of proteinuria at high altitudes. However, there is still insufficient clinical evidence to confirm the therapeutic effect of acetazolamide or ACEIs on renal pathological changes at high altitude. More basic and clinical research is needed to confirm the effect of high-altitude exposure on the kidneys as well as the efficacy of treatments.
